# Intergenerational genotypic interactions drive collective behavioural cycles in a social insect

**DOI:** 10.1098/rspb.2022.1273

**Published:** 2022-11-09

**Authors:** Stephanie L. Jud, Daniel Knebel, Yuko Ulrich

**Affiliations:** ^1^ Institute of Integrative Biology, ETHZ Zürich, Zürich 8092, Switzerland; ^2^ Max Planck Institute for Chemical Ecology, Hans-Knöll-Strasse 8, Jena 07745, Germany

**Keywords:** cycles, social insects, collective behaviour, developmental plasticity, cross-fostering, clonal raider ant

## Abstract

Many social animals display collective activity cycles based on synchronous behavioural oscillations across group members. A classic example is the colony cycle of army ants, where thousands of individuals undergo stereotypical biphasic behavioural cycles of about one month. Cycle phases coincide with brood developmental stages, but the regulation of this cycle is otherwise poorly understood. Here, we probe the regulation of cycle duration through interactions between brood and workers in an experimentally amenable army ant relative, the clonal raider ant. We first establish that cycle length varies across clonal lineages using long-term monitoring data. We then investigate the putative sources and impacts of this variation in a cross-fostering experiment with four lineages combining developmental, morphological and automated behavioural tracking analyses. We show that cycle length variation stems from variation in the duration of the larval developmental stage, and that this stage can be prolonged not only by the clonal lineage of brood (direct genetic effects), but also of the workers (indirect genetic effects). We find similar indirect effects of worker line on brood adult size and, conversely (but more surprisingly), indirect genetic effects of the brood on worker behaviour (walking speed and time spent in the nest).

## 1. Introduction

Cycles are ubiquitous across scales of biological organization, from cells contracting to generate cardiac rhythm [1] to entire animal populations migrating seasonally [2]. Social and gregarious animals often display collective activity cycles, which vary in period from seconds (e.g. fireflies flashing in unison [3,4]) to years (e.g. cicada brood emergence [5]). Collective cycling in turn requires behaviour to be synchronized across group members [6,7]. These synchronized behavioural oscillations can come about through two basic mechanisms: a global group wide variable that influences each group member or a local interaction between group members with no global connection among them [8].

An iconic collective cycle is that of army ants, whereby colonies made up of thousands or millions of individuals undergo stereotypical biphasic reproductive and behavioural cycles of about one month. This cycle closely tracks brood development: in the nomadic phase, colonies emigrate almost daily, hunting for prey to feed growing larvae. Larval pupation coincides with the entry into the statary phase, in which the queen lays eggs and the colony stays in one location [9,10]. While these iconic cycles have long fascinated naturalists [11–14], the basis of their regulation and maintenance cannot be probed experimentally, in part because most species displaying such cycles are notoriously difficult to keep under laboratory conditions.

The clonal raider ant (*Ooceraea biroi*) is a doryline ant displaying stereotypical army ant like colony cycles. Unlike army ants, however, this species is uniquely experimentally amenable. Colonies of the clonal raider ant are queenless and consist of genetically identical female workers that all reproduce synchronously, meaning that all colony members undergo reproductive and behavioural cycles. The colony cycle consists of alternating brood care and reproductive phases [15], characterized by the presence and absence of larvae, respectively [16–18]. In the brood care phase, workers nurse the larvae in the nest but also leave the nest to forage; in the reproductive phase in contrast, all workers stay in the nest to lay eggs, and no foraging takes place [17]. Brood stage controls colony phase: in the brood care phase—corresponding to the nomadic phase of army ant cycles—the presence of larvae suppresses worker ovarian activity and induces worker foraging activity [16,17]. When larvae become prepupae (i.e. shed their meconium and stop feeding), ovarian inhibition is released and workers synchronously lay eggs, while foraging activity stops. The colony switches back to the brood care phase when those eggs hatch into new larvae.

While brood stage controls cycle phase, it is unclear what controls cycle length. Although cycle length was initially assumed to be fixed [9], recent work has shown that it is highly plastic with respect to at least one group trait, colony size [18]. It is not known whether cycle length can vary with other basic colony features and in particular, whether it varies with the genotype of the colony. If cycle length varies with genotype, it is also unclear whether the genotype of the brood, of the workers, or both, drive the effect. In principle, cycle length could be controlled by the brood alone, if e.g. brood develops at a fixed, genetically determined speed, which in turn determines cycle length. However, because brood in social insects fully depend on workers for food and care, worker behaviour (e.g. foraging efficiency) is also likely to affect brood development. If worker behaviour varies across genotypes, then worker genotype could affect cycle length via effects on brood development.

In most social insects, brood–adult interactions are difficult to study experimentally due to the inherent complexity of colonies, which typically consist of a unique mix of one or more queens, workers of different ages and brood at different developmental stages. Consequently, the brood environment encountered by the workers, and the worker environment encountered by the brood are difficult to control and replicate. By contrast, asexual and synchronous [19] reproduction in the clonal raider ant provides precise control over the genetic background and the age of both workers and brood. Within a colony, workers belong to one of two subcastes that differ in morphology, behaviour and reproductive physiology: small, regular workers with two ovarioles and larger intercastes with four to six ovarioles, vestigial eyes and a tendency to spend more time in the nest [19–21]. Field collected clonal raider ant colonies belong to different clonal lineages (henceforth, ‘lines’) [22,23], which can readily be cross-fostered [21,24]. Differences in worker behaviour [21], anecdotal differences in brood developmental time [18], as well as differences in intercaste proportion [24] have been reported between two commonly used lines (A and B). Previous work on these two lines showed that the propensity of larvae to develop into intercastes is affected by epistatic interactions between larval and worker lines [24]. Furthermore, cross-fostering larvae of each line in colonies containing workers of both lines suggested that larvae of different lines vary in their effects on worker behaviour [21]. Collectively, this points to a role for larvae–worker genotypic interactions in driving colony cycle length.

Here, we use a cross-fostering approach to quantify the impact of genotypic interactions between larvae and workers on cycle length, worker behaviour and brood development in the clonal raider ant. We first establish that cycle length varies across lines using long-term data from laboratory-reared stock colonies. We then investigate the source of this variation by analysing the developmental and behavioural correlates of cycle length variation in a full-factorial cross-fostering experiment between four lines, combining survival, developmental and morphological analyses with automated behavioural tracking.

## 2. Material and methods

### (a) Cycle length variation across stock colonies

To test for baseline differences in cycle length between lines, we analysed long-term data (collected from October 2017 to May 2021; electronic supplementary material, table S1) on the total cycle length and the duration of each brood developmental stage (egg, larva, prepupa, pupa) in seven laboratory-reared stock colonies belonging to four lines (A, B, D, M) [22,23]. All stock colonies were maintained at 27 ± 1°C in airtight plastic containers with water-saturated plaster of Paris floors. During the brood care phase, stock colonies were fed with frozen ant (*Messor* or *Camponotus*) larvae and houseflies. Prior to September 2020, data were collected five times a week (from Monday to Friday) and thereafter, data were collected three times a week (on Mondays, Wednesdays and Fridays). If a change in brood developmental stage occurred on a day when data were not recorded, the initial stage was consistently assumed to last until the subsequent brood stage was observed. Only complete cycles (i.e. cycles in which the brood successfully developed from eggs to adults) were included in analyses. Cycle length was defined as the time (in days) elapsed between two successive larval hatching events.

### (b) Cross-fostering experiment

To remove sources of variation other than line, we used workers and larvae of the same age across all treatments. To obtain age-matched workers across all lines, we synchronized reproductive cycles across lines by separating 1000 workers from each of four stock colonies (C16, STC1, BG9, BG14) from different lines (A, B, D, M, respectively) to create four experimental colonies. Separated workers initiated a new cycle by laying eggs within 1–3 days. New workers eclosed from these eggs 26–28 days later (i.e. within 2 days of each other across experimental colonies) and were used as focal ants in the cross-fostering experiment. All focal ants were tagged with colour marks on the thorax and abdomen using oil-paint markers, so as to be individually recognized using automated tracking (see below). To obtain age-matched larvae across all lines, workers from the experimental colonies that had reared the focal ants were transferred to new nests (without brood) to initiate a new event of synchronized egg-laying. New eggs were laid by these workers within 4–5 days, which hatched into larvae on the same day across all lines (8–9 days after egg-laying).

Age-matched workers and larvae were used in a full-factorial cross-fostering experiment with 16 treatments (AA, AB, AD, AM, BA, etc. where the first letter of each treatment indicates worker line, and the second letter indicates brood line). Cross-fostering colonies were composed of eight age-matched (26 to 28 days old) focal ants and seven age-matched (5 days old) larvae housed in airtight Petri-dishes (5 cm in diameter) with a plaster of Paris floor. Colonies of this size show high fitness and normal behaviour in the clonal raider ant [18]. Each treatment was replicated six to eight times (depending on the availability of age-matched larvae and workers), resulting in 109 cross-fostering colonies in total. The colonies were placed randomly in a setup [18] equipped with cameras to record worker behaviour throughout the brood care phase (8–16 days, depending on the colony), itself housed in a climate room kept at 29 ± 1°C. Colonies were given 48 h to settle, after which any dead larvae were replaced, and the experiment started. For each colony, the experiment ended when all the original larvae had either eclosed into new adults or died. Every second day for the duration of the experiment, all colonies were cleaned (food debris and dead ants were removed), fed with frozen *Messor* pupae proportionally to estimated *O. biroi* larval biomass (one minor pupa for every third 3rd instar clonal raider ant larva and one major pupa for every third 4th instar clonal raider ant larva), and the plaster was humidified. Additionally, brood developmental stage and survival, as well as worker survival were quantified. At the end of the experiment, all newly eclosed adults (523 out of the original 763 larvae) were frozen at −80°C, and later used for dissections (to count ovariole number) and morphometric measurements (total body length, head with, gastral width and presence/absence of vestigial eyes, measured from images acquired with an Olympus Dual-Sensor Monochrome and Colour Camera (DP80) coupled to the software Olympus cellSens Standard (v.1.15; electronic supplementary material, figure S1). Individuals that had eyes and/or four or more ovarioles were categorized as intercastes.

### (c) Automated behavioural analyses

Videos were recorded for 10 min every 2 h throughout the brood care phase of each cross-fostering colony, starting 24 h after the start of the experiment. The positions of individual ants were extracted from videos using the automated tracking software anTraX v.1.0.2 [25]. Tracking performance was validated manually with the built-in procedure of anTraX, based on 100 automatically made assignments per camera. In social insect colonies, tasks are spatially segregated (e.g. nursing occurs in the nest whereas foraging occurs outside the nest) [26], meaning that spatial distribution can be used as a proxy for task performance [18,27]. We, therefore, used three spatial behavioural traits to characterize the tendency of individual ants to leave the nest to forage versus stay in the next with the brood: (i) proportion of time active (henceforth ‘activity’), defined as the proportion of frames in which an ant was moving at a speed greater than 1 mm s^−1^; (ii) walking speed, defined as the mean walking speed (in mm s^−1^) of an active ant, were both used as proxies for foraging activity. Activity and walking speed were averaged across videos for each ant; (iii) proportion of time in the nest, defined as the proportion of frames in which an ant was in the nest area, was used as a proxy for individual nursing behaviour. In ant colonies, workers cluster around their brood, and for each colony, the nest area (i.e. the brood pile) was thus identified as the area of densest worker presence, as follows: the area of the Petri dish was binned (80 × 80 bins), the normalized spatial distribution of all ants calculated, the two-dimensional histogram was square root transformed and smoothed with a Gaussian filter, and the nest area was defined as the 5% most populated bins. A threshold for minimal nest size (70 bins) was then applied to remove aberrant nests. Automatically detected nest areas were validated manually and incorrectly identified nests were removed so that only one nest per colony remained. This process was repeated every two consecutive movies (i.e. 20 min of videos every 4 h) for each colony, thereby capturing changes in the nest shape and position.

### (d) Statistical analyses

Statistical analyses were performed in R v.4.0.5 [28].

Kruskal–Wallis tests were used to compare cycle length and the duration of each brood developmental stage (eggs, larvae, prepupae, pupae) across stock colonies. Developmental stage durations were defined as the time (in days) elapsed between the appearance of a given brood stage (e.g. larvae) until the appearance of the next brood stage (e.g. prepupae). To compare cycle length and developmental stage durations between pairs of stock colonies, we conducted Dunn's tests (function *dunn_test* of package *rstatix*) with Benjamini–Hochberg adjustment for multiple testing.

In one cross-fostering colony (treatment AB), all workers died due to insufficient humidity and the colony was, therefore, excluded from all analyses. In one additional colony (treatment DA), all brood failed to develop into workers and this colony was thus excluded from analyses of developmental time, caste fate and body length, but retained in analyses of survival and worker behaviour. Furthermore, in one colony of each of the treatments AB, AD, BB, BD, DM and MA, the assignment rate of automated tracking was low (less than 60%) and these colonies were, therefore, excluded from behavioural analyses. After the exclusion of the seven above-mentioned colonies, an additional 29 individual ants were removed due to a low (less than 60%) assignment rate of automated tracking. After exclusion of these colonies and ants, automated tracking assigned 85.17% of the focal ant's locations with an error rate of less than or equal to 10%.

Unless stated otherwise, we used regression models (described below) to analyse the effects of worker line (a four-factor variable), brood line (a four-factor variable) and their interaction on all response variables in the cross-fostering experiment. In all models performed at the individual ant level, cross-fostering colony was used as a random effect to account for the non-independence of ants from the same colony. Model assumptions were verified using the function *simulateResiduals* of package *DHARMa.* Significance of model terms was assessed by sequential deletion of terms and model comparison (function *Anova* of package *car*). Terms that did not significantly contribute to model fit (*p* ≥ 0.05) were deleted and we report the results of reduced models here. If the interaction between brood and worker line was significant, pairwise comparisons of interest with user defined contrasts were conducted using the function *glht* of package *multcomp*. Comparisons of interest were those between pairs of treatments that shared either worker line (e.g. AB–AD) or brood line (BA–DA), as well as comparisons between pairs of control treatments (AA, BB, DD, MM). If the interaction was not significant, Tukey *post hoc* tests (function *glht* of package *multcomp*) were conducted between levels of significant main effects. In all cases, *p*-values were adjusted for multiple testing with the Benjamini–Hochberg method.

For each cross-fostering colony, larval stage duration was defined as the time (in days) elapsed between the start of the experiment (when larvae were 5- day old) and the appearance of the first prepupa. Pupal stage duration was defined as the time (in days) elapsed between the appearance of the first pupa and the eclosion of the first adult. Time to eclosion was defined as the time (in days) elapsed between the experiment start until the eclosion of the first adult. Linear models (LM, function *lm* from package *stats*) were used to analyse genotypic effects on time to eclosion and larval stage duration in the cross-fostering experiment. Time to eclosion and larval stage duration were rank transformed to satisfy model assumptions. Pupal stage duration data did not meet model assumptions and were therefore analysed using an aligned rank transform (ART) ANOVA (function *art* from package *ARTool*). The egg developmental time was not considered because the experiment began with larvae and the prepupal stage duration was not analysed because its mean duration (3.98 ± 0.24 days) was short compared to the data's temporal resolution (2 days).

Binomial generalized linear mixed models with Template Model Builder (glmmTMB, function *glmmTMB* of package *glmmTMB*) were used to analyse genotypic effects on worker and brood survival. Gaussian glmmTMB were used to analyse the behaviour (activity, proportion of time in the nest and walking speed) of individual focal ants. Activity was rank transformed and proportion of time in the nest was square transformed, both to better meet model assumptions. glmmTMBs for behavioural analyses did not fully meet model assumptions: quantile deviations were detected in the models for proportion of time in the nest and activity. Outliers were detected in models of walking speed and proportion of time in the nest; however, after removal of the outliers, the models remained qualitatively identical and thus outliers were retained in the final models.

A binomial glmmTMB was used to analyse the propensity of individual larvae to develop into intercastes or regular workers.

The three measurements of body size were highly correlated (Spearman's rank correlation tests: body length − head width: *S* = 4 737 960, *p* = 2.2 × 10^−16^; body length − abdomen width: *S* = 2 043 369, *p* = 2.2 × 10^−16^, head width − abdomen width: *S* = 4 920 246, *p* = 2.2 × 10^−16^) and thus only body length was analysed. An LMM (function *lmer* of package *lme4*) was used to analyse genotypic effects on individual body length.

Spearman's rank correlation tests were used to analyse the relationship between variables of interest at the colony level (larval stage duration versus intercaste proportion, larval stage duration versus mean body length).

## 3. Results

**Figure 1.**
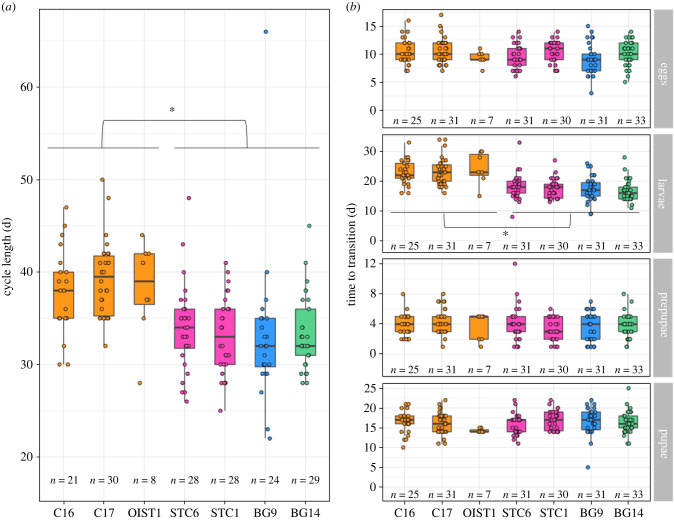
Colony cycles in stock colonies. Boxplots represent the median (bold horizontal line), the first and third quantiles (hinges) and the 95% confidence interval of the median (whiskers). Data points and sample sizes represent colony cycles analysed per stock colony. Colours indicate line (orange: A, pink: B, blue: D, green: M). *p < 0.05. (*a*) Total cycle length. (*b*) Duration of each brood developmental stage. (Online version in colour.)

Cycle length varied across stock colonies (Kruskal–Wallis rank sum test, *χ*^2^ = 48.09, d.f. = 6, *p* = 1.13 × 10^−8^; figure 1*a*), with almost a week difference between the longest (colony C17: mean ± s.e. 39.23 ± 0.74 days) and the shortest (colony BG9: 33 ± 1.66 days) mean cycle lengths. Mean cycle length in all three stock colonies of line A was longer than in the four stock colonies belonging to other lines (Dunn's test, C16, C17, OIST1 versus STC6, STC1, BG9, BG14, all *p* < 4.3 × 10^−2^; electronic supplementary material, table S2). Breaking down the cycle in different brood developmental stages revealed that differences in cycle length stemmed from differences in larval stage duration between stock colonies (*χ*^2^ = 65.24, d.f. = 6, *p* = 3.85 × 10^−12^; figure 1*b*), and not from differences in the duration of the egg, prepupal or pupal stages (eggs: *χ*^2^ = 10.69, d.f. = 6, *p* = 0.1; prepupae: *χ*^2^ = 4.96, d.f. = 6, *p* = 0.55; pupae: *χ*^2^ = 10.18, d.f. = 6, *p* = 0.12). Indeed, larvae of all three stock colonies of line A took longer to develop than larvae of all other stock colonies from different lines (Dunn's test, C16, C17, OIST1 versus STC6, STC1, BG9, BG14, all *p* < 1.11 × 10^−2^; electronic supplementary material, table S2). Thus, variation in cycle length across stock colonies is associated with clonal lineage and arises from variation in larval stage duration.

In the cross-fostering experiment, both worker survival (mean ± s.e.: 0.92 ± 0.01) and brood survival (0.68 ± 0.02) were consistently high across treatments. Neither worker survival nor larval survival were affected by worker line (glmmTMB, worker survival: *χ*^2^ = 0.56, d.f. = 3, *p* = 0.91; brood survival: *χ*^2^ = 1.59, d.f. = 3, *p* = 0.66; electronic supplementary material, figure S2), brood line (worker survival: *χ*^2^ = 2.08, d.f. = 3, *p* = 0.56; brood survival: *χ*^2^ = 2.45, d.f. = 3, *p* = 0.48) or their interaction (worker survival: *χ*^2^ = 7.16, d.f. = 9, *p* = 0.62; brood survival: *χ*^2^ = 9.91, d.f. = 9, *p* = 0.35). The lack of effect of worker line on brood survival supports the view that workers cannot differentiate or do not favour brood of their own line over brood of other lines.

**Figure 2.**
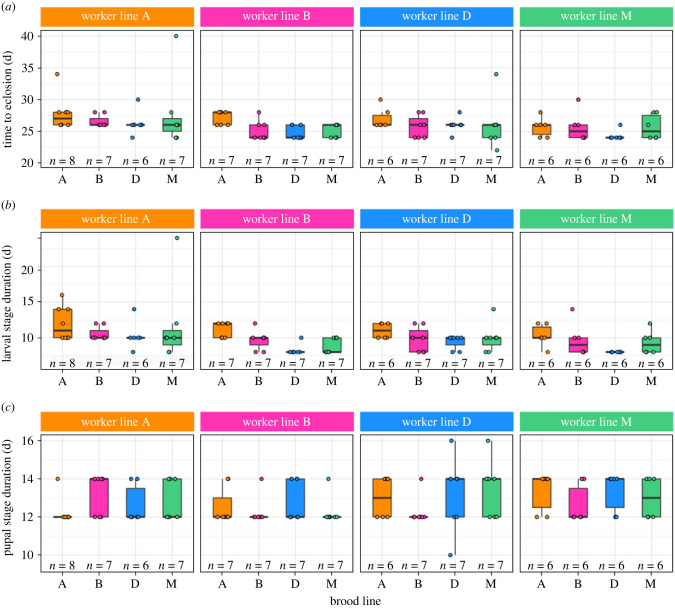
Colony cycles in cross-fostered colonies. Boxplots represent the median (bold horizontal line), the first and third quantiles (hinges) and 95% confidence interval of the median (whiskers). Data points and sample sizes indicate replicate colonies. Colours indicate lines (orange: A, pink: B, blue: D, green: M). (*a*) Time to eclosion as a function of worker and brood lines. (*b*) Larval stage duration as a function of worker and brood lines. (*c*) Pupal stage duration as a function of worker and brood lines. (Online version in colour.)

Brood time to eclosion was affected by both worker line (LM, *F*_3,100_ = 3.71, *p* = 1.41 × 10^−2^) and brood line (*F*_3,100_ = 4.69, *p* = 4.16 × 10^−3^; figure 2*a*). Brood of line A, irrespective of the worker line rearing them, took longer to develop into adults than brood of other lines (*post hoc* tests: A versus B, D, M, all *p* < 2.41 × 10^−2^; electronic supplementary material, table S3). Additionally, brood reared by workers of line A, irrespective of their own line, took longer to develop into adults than brood reared by workers of line M (M–A: *t* = −3.12, *p* = 1.44 × 10^−2^; figure 2*a*). As in stock colonies (figure 1), variation in developmental time across cross-fostering colonies was driven by variation in larval stage duration. Larval stage duration was affected by both worker line (LM, *F*_3,100_ = 4.81, *p* = 3.56 × 10^−3^) and brood line (*F*_3,100_ = 10.62, *p* = 4 × 10^−6^; figure 2*b*). Larvae of line A took longer to become prepupae than larvae of all other lines (*post hoc* tests, A versus B, D, M, all *p* < 7.7 × 10^−3^; electronic supplementary material, table S3) and larvae of line B took longer to develop than larvae of line D (D–B: *t* = −2.54, *p* = 1.89 × 10^−2^), irrespective of the line of the workers rearing them. In addition, larvae took longer to become prepupae when reared by workers of line A than by workers of lines B and M (B–A: *t* = −2.88, *p* = 1.47 × 10^−2^; M–A, *t* = −3.44, *p* = 5.06 × 10^−3^). Unlike the larval stage, the duration of the pupal stage did not vary with either brood line, worker line or their interaction (ART, worker line: *f*_3,91_ = 1.39, *p* = 0.25; brood line: *f*_3,91_ = 0.72, *p* = 0.54; interaction: *f*_9,91_ = 0.87, *p* = 0.56; figure 2*c*). Thus, variation in cycle length arises from variation in larval stage duration and is determined by both larval and worker lines. In line with this, colonies composed of line A workers and line A larvae had the longest larval stage (12 ± 0.85 days) across treatments.

**Figure 3.**
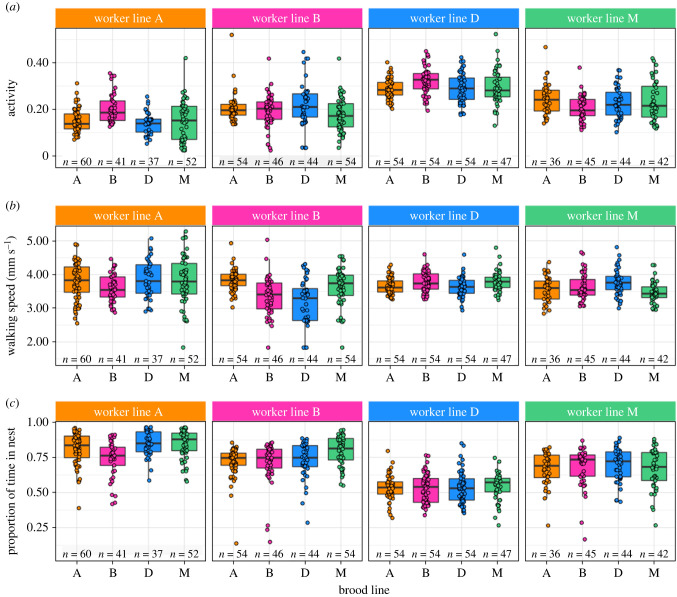
Worker behaviour in cross-fostered colonies. Boxplots show the median (bold horizontal line), the first and third quantiles (hinges), and the 95% confidence interval of the median (whiskers). Points and sample sizes represent individual workers. Colours indicate lines (orange: A, pink: B, blue: D, green: M). (*a*) Activity as a function of brood and worker lines. (*b*) Walking speed as a function of brood and worker lines. (*c*) Proportion of time in the nest as a function of brood and worker lines. (Online version in colour.)

Because larvae rely on food and care provided by workers to develop, we next asked whether differences in cycle length were associated with differences in worker behaviour. Worker activity—a proxy for foraging behaviour—was affected by the workers’ own line (glmmTMB, *χ*^2^ = 149.25, d.f. = 3, *p* < 2.2 × 10^−16^; figure 3*a*). Apart from one comparison (*post hoc* tests, M–B, *z* = 2.29, *p* = 0.1) the activity of all worker lines differed from each other (D > M, B > A, all *p* < 1.27 × 10^−2^; electronic supplementary material, table S4).

Another proxy for foraging, worker walking speed was influenced by an interaction between worker line and brood line (glmmTMB, *χ*^2^ = 18.9, d.f. = 9, *p* = 2.6 × 10^−2^; figure 3*b*). Pairwise comparisons revealed that B workers rearing D larvae walked slower than workers of two other lines rearing the same larvae (*post hoc* tests, BD–AD: *z* = −3.44, *p* = 1.55 × 10^−2^; MD–BD: *z* = 3.21, *p* = 2.36 × 10^−2^; electronic supplementary material, table S5) and, more strikingly, that they also walked slower than identical workers rearing A larvae (BD–BA, *z* = 3.68, *p* = 1.24 × 10^2^).

The proportion of time workers spent in the nest—a proxy for nursing behaviour—was affected by the workers' line (glmmTMB, *χ*^2^ = 226.83, d.f. = 3, *p* < 2.2 × 10^−16^; figure 3*c*) and by the line of the larvae they were rearing (*χ*^2^ = 8.47, d.f. = 3, *p* = 3.72 × 10^−2^). All worker lines differed in the proportion of time they spent in the nest (*post hoc* tests, A > B > M > D, all *p* < 9.12 × 10^−3^; electronic supplementary material, table S6). Furthermore, workers that reared brood of line M spent more time in the nest than workers that reared brood of line B (M–B: *z* = 2.74, *p* = 3 × 14^−2^).

Thus, worker behaviour was not only affected by their own genotype, but also by the genotype of the larvae they reared.

**Figure 4.**
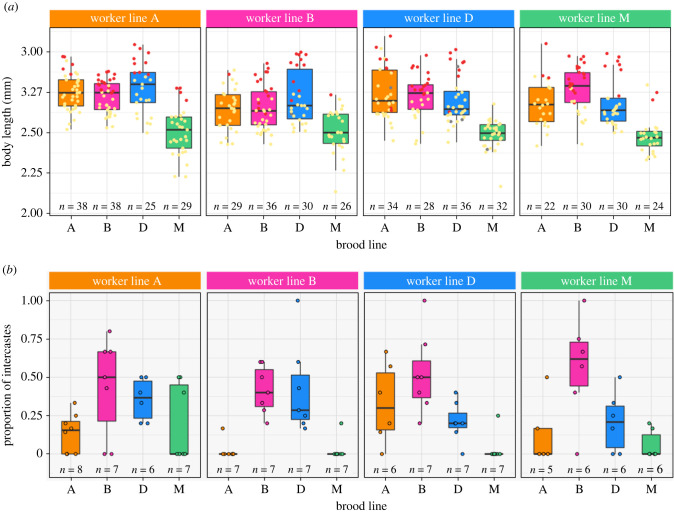
Caste fate and body length in the cross-fostering experiment. Boxplots represent the median (bold horizontal line), the first and third quantiles (hinges) and the 95% confidence interval of the median (whiskers). Colours indicate lines (orange: A, pink: B, blue: D, green: M). (*a*) Body length in adults eclosed from cross-fostered larvae as a function of worker line and brood line. Sample sizes and data points represent individuals classified as intercastes (red), regular workers (yellow) or undetermined (grey). (*b*) Proportion of intercastes as a function of worker line and brood line. Sample sizes and data points represent colonies. (Online version in colour.)

Because developmental plasticity is often linked to caste in social insects, we then asked whether differences in brood developmental time affected the morphology (caste, body size) of the resulting adults (figure 4*a*).

We found substantial variation in caste fate across treatments, both within and between lines, showing extensive developmental plasticity in this system. The proportion of intercastes ranged from 2.38 ± 2.38% in treatment BA to 56.47 ± 13.91% in treatment MB (figure 4*b*). Intercaste proportion was influenced by an interaction between worker and brood lines (glmmTMB: *χ*^2^ = 17.51, d.f. = 9, *p* = 4.14 × 10^−2^). Differences in intercaste production stemmed from the propensity for brood of line B to develop into intercastes more readily than brood of other lines, even when reared by the same workers (*post hoc* tests, AB–AA, *z* = 2.89, *p* = 4.19 × 10^−2^, DM–DB, *z* = −3.18, *p* = 2.24 × 10^−2^, MB–MA, *z* = 3.27, *p* = 2.24 × 10^−2^, MD–MB, *z* = −3.15, *p* = 2.24 × 10^−2^, MM–MB, *z* = −3.58, *p* = 1.88 × 10^−2^; electronic supplementary material, table S7 and figure S3*a*).

Adult body length was affected by an interaction between worker and brood lines (LMM, *χ*^2^ = 19.16, d.f. = 9, *p* = 2.39 × 10^−2^; figure 4*a*). Control colonies of line A produced larger workers than control colonies of line B (*post hoc* tests, BB–AA, *z* = −2.86, *p* = 1.26 × 10^−2^) and control colonies of line M produced smaller workers than all other control colonies (MM versus AA, BB, DD: all *p* < 1.12 × 10^−4^; electronic supplementary material, table S8). Most other differences across cross-fostered colonies stemmed from the propensity for brood of line M to grow into smaller adults than brood of other lines, even when reared by the same workers (AM–AA, AM–AB, AM–AD, BM–BA, BM–BB, BM–BD, DM–DA, DM–DB, DM–DD, MM–MA, MM–MB and MM–MD : *p* < 4.81 × 10^−2^; electronic supplementary material, table S8 and figure S3*b*). Brood line influenced adult body length in only one other case (MD–MB: *z* = −2.35, *p* = 4.81 × 10^−2^). Interestingly, worker line also affected the adult size of the brood they reared. In two cases, A workers produced larger adults than other workers from the same larvae (*post hoc* tests, BA–AA, *z* = −3, *p* = 8.55 × 10^−3^; MD–AD, *z* = −2.51, *p* = 3.26 × 10^−2^), while in two other cases, B workers produced smaller adults than other workers from otherwise identical larvae (DA–BA, *z* = 2.81, *p* = 1.41 × 10^−2^; MB–BB, *z* = 3.09, *p* = 6.84 × 10^−3^). Thus, while adult morphology was influenced primarily by the brood's own line, we also detected indirect genetic effects arising from the rearing environment (worker line). Despite variation in brood developmental time and adult morphology, we find no clear relationship between these two traits across colonies (Spearman's rank correlation tests: larval stage duration − intercaste proportion: *S* = 188 903, *p* = 0.19; larval stage duration − body length: *S* = 139 478, *p* = 0.175).

## 4. Discussion

Using long-term data and a cross-fostering experiment, we show that the duration of a collective cycle varies across clonal lineages in a social insect. We find that differences in cycle length stem from variation in the duration of a specific brood developmental stage—the larval stage—and that the duration of that stage is influenced not only by the genotype of the brood itself, but also by the genotype of the workers rearing them. Larvae are the only feeding brood developmental stage, and *O. biroi* workers only forage in the presence of larvae [16], indicating a role for nutrient acquisition and processing in driving genotypic effects on cycle length. In line with this, the worker line that consistently prolonged larval development (A) had the lowest activity and highest time spent in the nest, both of which point to reduced foraging activity. This is supported by Piekarski *et al*. [29], who report reduced foraging activity in workers of line A. The finding that both larvae and workers of the same line (A) were sufficient to prolong cycle length suggests a genetic effect acting on both larval growth and worker foraging activity. Such an effect could stem, for example, from a lower metabolic rate in line A.

We find pervasive direct genetic effects on brood development (the line of the brood affected its developmental time, caste and adult body size) and on worker behaviour (the line of workers affected their activity and time spent in the nest). Additionally, we detect indirect genetic effects of workers on brood development (worker line affected brood developmental time and final size) likely reflecting the dependence of larvae on workers for food and care. Some of these findings are corroborated by Piekarski *et al*. [29] who report higher motility in line D workers and larger body size in brood reared by workers of line A using different metrics. More surprisingly, we detect several instances of indirect genetic effects of the brood on worker behaviour, with larval genotype affecting the amount of time workers spend in the nest as well as their walking speed. While effects of the rearing environment on brood development, and particularly on sex and caste allocation, have been extensively studied in social insects [29–33], whether and how the brood can affect worker behaviour has received comparatively little attention. Previous work in other systems has shown that the brood of social insects can produce chemical [34], behavioural [35] and acoustic [36] cues, some of which influence worker behaviour [37,38]. For example, honeybee larvae secrete a pheromone that affects worker physiology and behaviour [37,39,40]. Similarly, some ant larvae can increase their own food provisioning through body movements [35,41], akin to begging behaviour in other systems [42]. Our findings indicate that clonal raider ant larvae produce a global cue affecting worker behaviour, and that the intensity (or nature) of this cue varies across clonal lineages, as has recently been theoretically predicted [21]. They also illustrate how the brood—often overlooked colony members in social insects—can play an active role in the regulation of colony behaviour, suggesting that larvae may be able to affect their own developmental trajectory by influencing worker foraging activity.

Genotypic interactions between brood ‘begging’ intensity and worker foraging activity would in turn be expected to have far-reaching consequences on colony growth, composition and behaviour over time. We find substantial plasticity in the production of intercastes stemming from genotypic interactions between the brood and workers. Previous work with two commonly used lines [24] proposed that such plasticity reflects different reproductive strategies across lines. In particular, a line with high intercaste production was proposed to be favoured in environments where line-mixing occurs frequently. This is because by producing offspring that reproduce more and forage less, that line could outcompete the other in mixed colonies, akin to socially parasitic lineages of parthenogenetic ants [43] and bees [44]. Here, brood of line B developed into intercastes at a higher frequency than all other lines and would, therefore, be expected to outcompete these other lines in mixed colonies over time, supporting the view that it may act as a general facultative social parasite in the presence of other lines.

Our finding of extant direct genetic effects and bi-directional indirect genetic effects between brood and adults is reminiscent of classic findings in other systems [45–47], where genetically based variation in brood solicitation and adult provisioning are thought to drive complex coevolutionary dynamics [48,49]. While brood care is central to the biology and the evolution of social insects, these questions have received comparatively little attention in social insects, in part due to practical limitations linked to the complexity of colonies. The clonal raider ant, with its comparatively simple social organization (queenlessness, asexual reproduction), relatively short generation time and unique experimental amenability is a promising system to explore both the ultimate and proximate bases of brood–adult interactions in social insects.

## Data Availability

Data available from the Dryad Digital Repository: https://doi.org/10.5061/dryad.n02v6wx0v [50]. The data are provided in electronic supplementary material [51].

## References

[1] Fukuta H, Little WC. 2008 The cardiac cycle and the physiologic basis of left ventricular contraction, ejection, relaxation, and filling. Heart Fail. Clin. **4**, 1-11. (10.1016/j.hfc.2007.10.004) 18313620 PMC2390899

[2] Gwinner E. 1977 Circannual rhythms in bird migration. Annu. Rev. Ecol. Syst. **8**, 381-405. (10.1146/annurev.es.08.110177.002121)

[3] Buck J, Buck E. 1966 Biology of synchronous flashing of fireflies. Nature **211**, 562-564. (10.1038/211562a0)

[4] Sarfati R, Hayes JC, Peleg O. 2021 Self-organization in natural swarms of *Photinus carolinus* synchronous fireflies. Sci. Adv. **7**, eabg9259. (10.1126/sciadv.abg9259) 34233879 PMC8262802

[5] Williams KS, Simon C. 1995 The ecology, behavior, and evolution of periodical cicadas. Annu. Rev. Entomol. **40**, 269-295. (10.1146/annurev.en.40.010195.001413)

[6] Chiara V, Arrufat P, Jeanson R. 2022 A variable refractory period increases collective performance in noisy environments. Proc. Natl Acad. Sci. USA **119**, e2115103119. (10.1073/pnas.2115103119) 35254873 PMC8944924

[7] Camazine S, Deneubourg J-L, Franks NR, Sneyd J, Theraula G, Bonabeau E. 2001 Self-organization in biological systems. Princeton, NJ: Princeton University Press.

[8] Cole BJ, Trampus FI. 1999 Activity cycles in ant colonies: worker interactions and decentralized control. In Information processing in social insects (eds C Detrain, JL Deneubourg, JM Pasteels), pp. 289-307. Basel, Switzerland: Birkhäuser.

[9] Kronauer DJC. 2009 Recent advances in army ant biology (Hymenoptera: Formicidae). Myrmecol News **12**, 51-65.

[10] Kronauer DJC. 2020 Army ants. Cambridge, MA: Harvard University Press.

[11] Schneirla TC. 1971 Army ants: a study in social organization. San Francisco, CA: WH Freeman and Company.

[12] Schneirla TC, Brown RZ. 1950 Army-ant life and behavior under dry-season conditions. 4. Further investigation of cyclic processes in behavioral and reproductive functions. Bull. Am. Mus. Nat. Hist. **95**, 265-353.

[13] Wilson EO. 1971 The insect societies. Cambridge, MA: Harvard University Press.

[14] Teles Da Silva M. 1977 Behavior of the army ant *Eciton burchelli* Westwood (Hymenoptera Formicidae) in the Belem region. II. Bivouacs. Bolm. Zool. Univ. S. Paulo **2**, 107-128.

[15] Ravary F, Jaisson P. 2002 The reproductive cycle of thelytokous colonies of *Cerapachys biroi* Forel (Formicidae, Cerapachyinae). Insectes Soc. **49**, 114-119. (10.1007/s00040-002-8288-9)

[16] Ravary F, Jahyny B, Jaisson P. 2006 Brood stimulation controls the phasic reproductive cycle of the parthenogenetic ant *Cerapachys biroi*. Insectes Soc. **53**, 20-26. (10.1007/s00040-005-0828-7)

[17] Ulrich Y, Burns D, Libbrecht R, Kronauer DJC. 2016 Ant larvae regulate worker foraging behavior and ovarian activity in a dose-dependent manner. Behav. Ecol. Sociobiol. **70**, 1011-1018. (10.1007/s00265-015-2046-2) 27616809 PMC5015688

[18] Ulrich Y, Saragosti J, Tokita CK, Tarnita CE, Kronauer DJC. 2018 Fitness benefits and emergent division of labour at the onset of group living. Nature **560**, 635-638. (10.1038/s41586-018-0422-6) 30135576 PMC6121774

[19] Ravary F, Jaisson P. 2004 Absence of individual sterility in thelytokous colonies of the ant *Cerapachys biroi* Forel (Formicidae, Cerapachyinae). Insectes Soc. **51**, 67-73. (10.1007/s00040-003-0724-y)

[20] Lecoutey E, Châline N, Jaisson P. 2011 Clonal ant societies exhibit fertility-dependent shifts in caste ratios. Behav. Ecol. **22**, 108-113. (10.1093/beheco/arq182)

[21] Ulrich Y, Kawakatsu M, Tokita CK, Saragosti J, Chandra V, Tarnita CE, Kronauer DJC. 2021 Response thresholds alone cannot explain empirical patterns of division of labor in social insects. PLoS Biol. **19**, 1-21. (10.1371/journal.pbio.3001269) PMC8211278 34138839

[22] Kronauer DJC, Pierce NE, Keller L. 2012 Asexual reproduction in introduced and native populations of the ant *Cerapachys biroi*. Mol. Ecol. **21**, 5221-5235. (10.1111/mec.12041) 23013522

[23] Trible W, McKenzie SK, Kronauer DJC. 2020 Globally invasive populations of the clonal raider ant are derived from Bangladesh: source of the clonal raider ant invasion. Biol. Lett. **16**, 1-5. (10.1098/rsbl.2020.0105) PMC7336853 32544382

[24] Teseo S, Châline N, Jaisson P, Kronauer DJC. 2014 Epistasis between adults and larvae underlies caste fate and fitness in a clonal ant. Nat. Commun. **5**, 1-8. (10.1038/ncomms4363) 24561920

[25] Gal A, Saragosti J, Kronauer DJC. 2020 Antrax, a software package for high-throughput video tracking of color-tagged insects. Elife **9**, 1-32. (10.7554/eLife.58145) PMC7676868 33211008

[26] Mersch DP, Crespi A, Keller L. 2013 Tracking individuals shows spatial fidelity is a key regulator of ant social organization. Science **340**, 1090-1093. (10.5061/dryad.2pm42) 23599264

[27] Sendova-Franks AB, Franks NR. 1995 Spatial relationships within nests of the ant *Leptothorax unifasciatus* (Latr.) and their implications for the division of labour. Anim. Behav. **50**, 121-136. (10.1006/anbe.1995.0226)

[28] R Core Team. 2021 R: a language and environment for statistical computing. Vienna, Austria: R Foundation for Statistical Computing.

[29] Piekarski PK, Valdés-Rodríguez S, Kronauer DJC. 2022 Conditional indirect genetic effects of caregivers on offspring growth and development in the clonal raider ant. Unpublished manuscript. 10.1093/beheco/arad033 PMC10332452 37434637

[30] Bourke AFG, Ratnieks FLW. 1999 Kin conflict over caste determination in social Hymenoptera. Behav. Ecol. Sociobiol. **46**, 287-297. (10.1007/s002650050622)

[31] Linksvayer TA. 2006 Direct, maternal, and sibsocial genetic effects on individual and colony traits in an ant. Evolution (NY) **60**, 2552-2561. (10.1554/06-011.1) 17263116

[32] Reuter M, Keller L. 2001 Sex ratio conflict and worker production in eusocial Hymenoptera. Am. Nat. **158**, 166-177. (10.1086/321311) 18707345

[33] Warner MR, Lipponen J, Linksvayer TA. 2018 Pharaoh ant colonies dynamically regulate reproductive allocation based on colony demography. Behav. Ecol. Sociobiol. **72**, 1-3. (10.1007/s00265-017-2430-1)

[34] den Boer SPA, Duchateau MJHM. 2006 A larval hunger signal in the bumblebee *Bombus terrestris*. Insectes Soc. **53**, 369-373. (10.1007/s00040-006-0883-8)

[35] Kaptein N, Billen J, Gobin B. 2005 Larval begging for food enhances reproductive options in the ponerine ant *Gnamptogenys striatula*. Anim. Behav. **69**, 293-299. (10.1016/j.anbehav.2004.04.012)

[36] Casacci LP, Thomas JA, Sala M, Treanor D, Bonelli S, Balletto E, Schönrogge K. 2013 Ant pupae employ acoustics to communicate social status in their colony's hierarchy. Curr. Biol. **23**, 323-327. (10.1016/j.cub.2013.01.010) 23394832

[37] Schultner E, Oettler J, Helanterä H. 2017 The role of brood in eusocial Hymenoptera. Q. Rev. Biol. **92**, 39-78. (10.1086/690840) 29558609

[38] Mas F, Kölliker M. 2008 Maternal care and offspring begging in social insects: chemical signalling, hormonal regulation and evolution. Anim. Behav. **76**, 1121-1131. (10.1016/j.anbehav.2008.06.011)

[39] le Conte Y, Bécard JM, Costagliola G, de Vaublanc G, el Maâtaoui M, Plettner D, Crauser E, Slessor KN. 2006 Larval salivary glands are a source of primer and releaser pheromone in honey bee (*Apis mellifera L.*). Naturwissenschaften **93**, 237-241. (10.1007/s00114-006-0089-y) 16541233

[40] Slessor KN, Winston ML, le Conte Y. 2005 Pheromone communication in the honeybee (*Apis mellifera L.*). J. Chem. Ecol. **31**, 2731-2745. (10.1007/s10886-005-7623-9) 16273438

[41] Creemers B, Billen J, Gobin B. 2003 Larval begging behaviour in the ant *Myrmica rubra*. Ethol. Ecol. Evol. **15**, 261-272. (10.1080/08927014.2003.9522671)

[42] Ottosson U, Bäckman J, Smith HG. 1997 Begging affects parental effort in the pied flycatcher, *Ficedula hypoleuca*. Behav. Ecol. Sociobiol. **41**, 381-384. (10.1007/s002650050399)

[43] Dobata S, Tsuji K. 2013 Public goods dilemma in asexual ant societies. Proc. Natl Acad. Sci. USA **110**, 16 056-16 060. (10.1073/pnas.1309010110) PMC3791774 24046364

[44] Martin SJ, Beekman M, Wossler TC, Ratnieks FLW. 2022 Parasitic Cape honeybee workers, *Apis mellifera capensis*, evade policing. Nature **415**, 163-165. (10.1038/415163a) 11805832

[45] Hager R, Johnstone RA. 2003 The genetic basis of family conflict resolution in mice. Nature **421**, 533-535. (10.1038/nature01239) 12556892

[46] Agrawal AF, Brodie III ED, Brown J. 2001 Parent-offspring coadaptation and the dual genetic control of maternal care. Science **292**, 1710-1712. (10.1126/science.1059910) 11387474

[47] Lock JE, Smiseth PT, Moore AJ. 2004 Selection, inheritance, and the evolution of parent-offspring interactions. Am. Nat. **164**, 13-24. (10.1086/421444) 15266367

[48] Kölliker M. 2005 Ontogeny in the family. Behav. Genet. **35**, 7-18. (10.1007/s10519-004-0852-9) 15674529

[49] Kölliker M, Richner H. 2001 Parent-offspring conflict and the genetics of offspring solicitation and parental response. Anim. Behav. **62**, 395-407. (10.1006/anbe.2001.1792)

[50] Jud SL, Knebel D, Ulrich Y. 2022 Data from: Intergenerational genotypic interactions drive collective behavioural cycles in a social insect. Dryad Digital Repository. (10.5061/dryad.n02v6wx0v) PMC9627708 36321497

[51] Jud SL, Knebel D, Ulrich Y. 2022 Intergenerational genotypic interactions drive collective behavioural cycles in a social insect. Figshare. (10.6084/m9.figshare.c.6251416) PMC9627708 36321497

